# Supplementary material for: A novel vaccine strategy using quick and easy conversion of bacterial pathogens to unnatural amino acid-auxotrophic suicide derivatives
Source: Microbiol Spectr. 2024 Feb 22;12(4):e03557-23. doi: 10.1128/spectrum.03557-23 (PMC10986568; doi:10.1128/spectrum.03557-23)
Supplement: Supplemental material — Figures S1 to S6; Tables S1 and S2. [file spectrum.03557-23-s0001.pdf]

Supplementary information

A novel vaccine strategy using quick and easy conversion of bacterial pathogens to unnatural amino acid-auxotrophic suicide derivatives

Yuya Nagasawa[1,4], Momoko Nakayama[2,4], Yusuke Kato[3,4,*], Yohsuke Ogawa[1], Swarmistha Devi Aribam[2], Yusaku Tsugami[1], Taketoshi Iwata[2], Osamu Mikami[1], Aoi Sugiyama[1], Megumi Onishi[1], Tomohito Hayashi[1,5,*], Masahiro Eguchi[2,*],

[1]National Institute of Animal Health, National Agriculture and Food Research Organization (NARO), Sapporo, Hokkaido, Japan
[2]National Institute of Animal Health, National Agriculture and Food Research Organization (NARO), Tsukuba, Ibaraki, Japan
[3]Institute of Agrobiological Sciences, National Agriculture and Food Research Organization (NARO), Tsukuba, Ibaraki, Japan
[4]First three authors equally contributed to this work.
[5]Present affiliation: Nihon Zenyaku Kogyo, Co., Ltd., Koriyama, Fukushima, Japan

*Correspondence:
Yusuke Kato (kato@affrc.go.jp)
Tomohito Hayashi (hayashi-tomohito@zenoaq.jp)
Masahiro Eguchi (egumaro@affrc.go.jp)

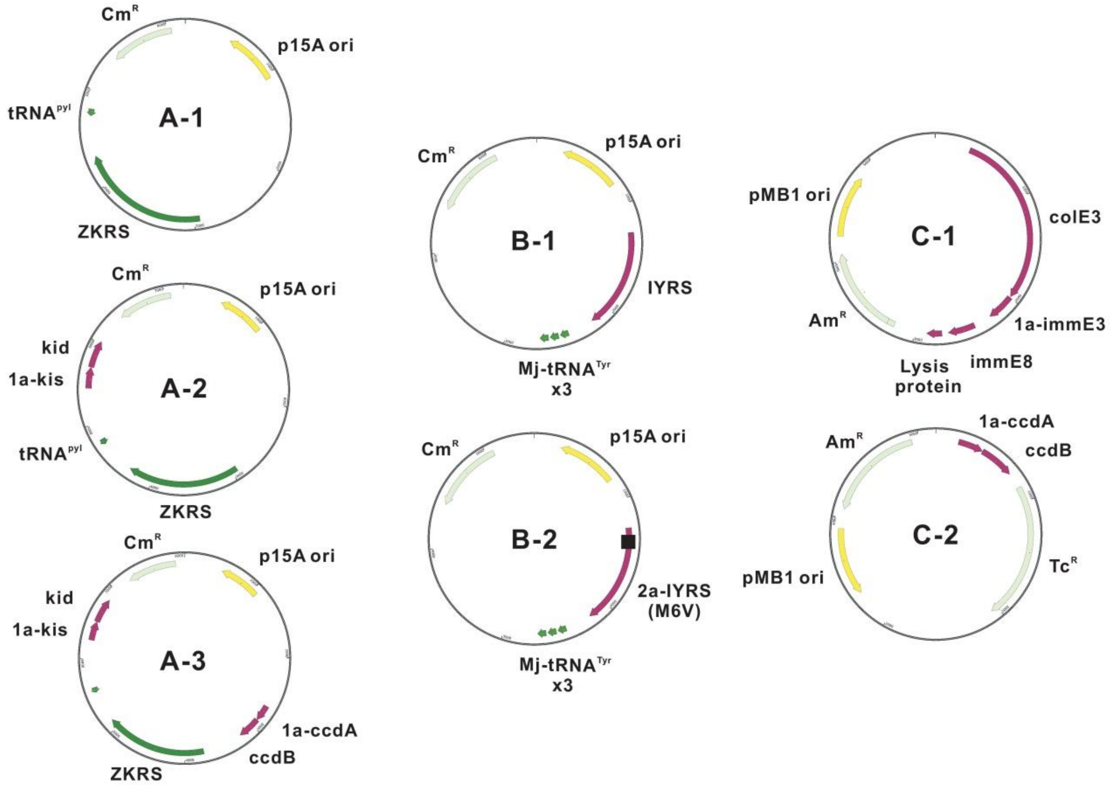

Supplementary Figure 1 Plasmid maps.

The plasmids used to construct the uAA-auxotrophic suicidal bacterial vaccines are shown. Plasmid A and B contain the p15A origin and the ZK or IY incorporation system consisting of a specific aminoacyl-tRNA synthetase and its cognate $tRNA_{CUA}$. Although A-1 contains only the ZK incorporation system, A-2 and A-3 also carry *kid-kis* and both *kid-kis* and *ccdB-ccdA,* respectively. A TAG stop codon sequence, abbreviated as 1a, was inserted next to the translation start codon of antitoxin genes. Whereas B-1 maintains the intact IY incorporation system, B-2 contains a modified system to reduce leakage expression in the absence of IY using a positive-feedback loop, 2a-IYRS(M6V) (42). C-1 and C-2 carry the toxin-antitoxin gene *colE3-immE3* and *ccdB-ccdA* with the TAG insertion, respectively. Plasmid C is a pMB1 origin plasmid which can be co-transformed with plasmid A or B.

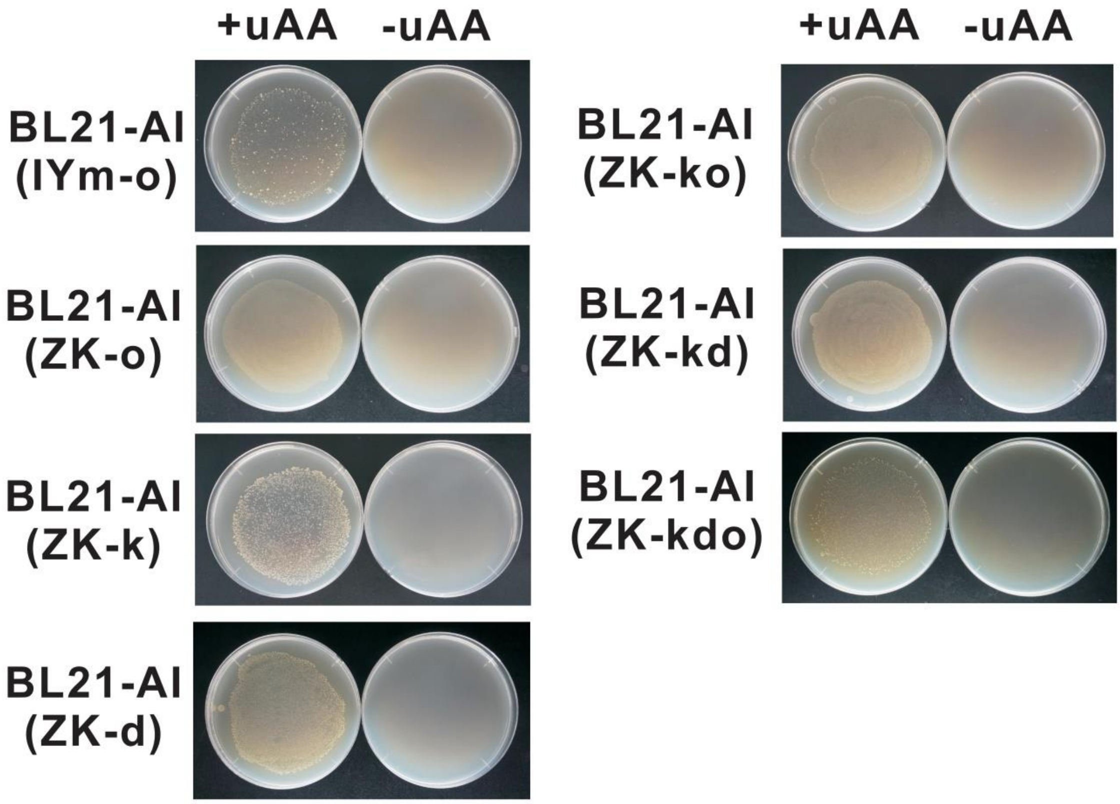

Supplementary Figure 2 The uAA-auxotrophic *E. coli* BL21-AI laboratory strains.

Various combination of plasmids shown in Supplementary Figure 1 were transfected into BL21-AI. Growth was evaluated in the presence and absence of 1 mM IY for BL21-AI(IYm-o) or 3 mM ZK for other strains.

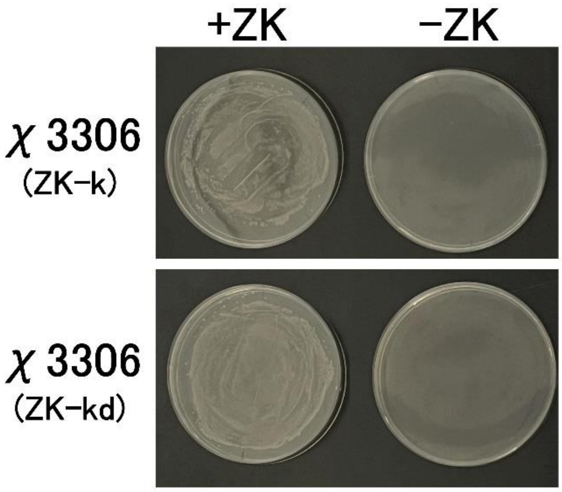

Supplementary Figure 3 The uAA-auxotrophic *S. enterica* strains.

S. enterica χ3306 (ZK-k) and χ3306 (ZK-kd) harbor plasmids A-2 and A-3, respectively. Growth was evaluated in the presence and absence of 3 mM ZK.

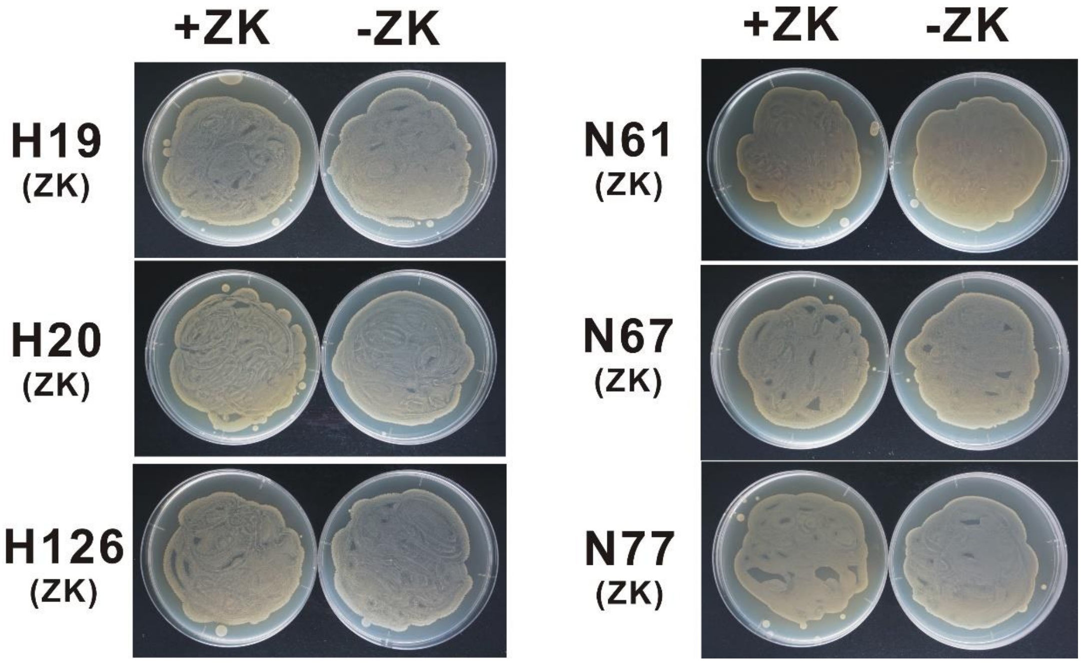

Supplementary Figure 4 ***E. coli*** **clinical isolates were tolerant for ZK-incorporation.**

Plasmid A-1 was transfected. Growth was tested in the presence and absence of 3 mM ZK.

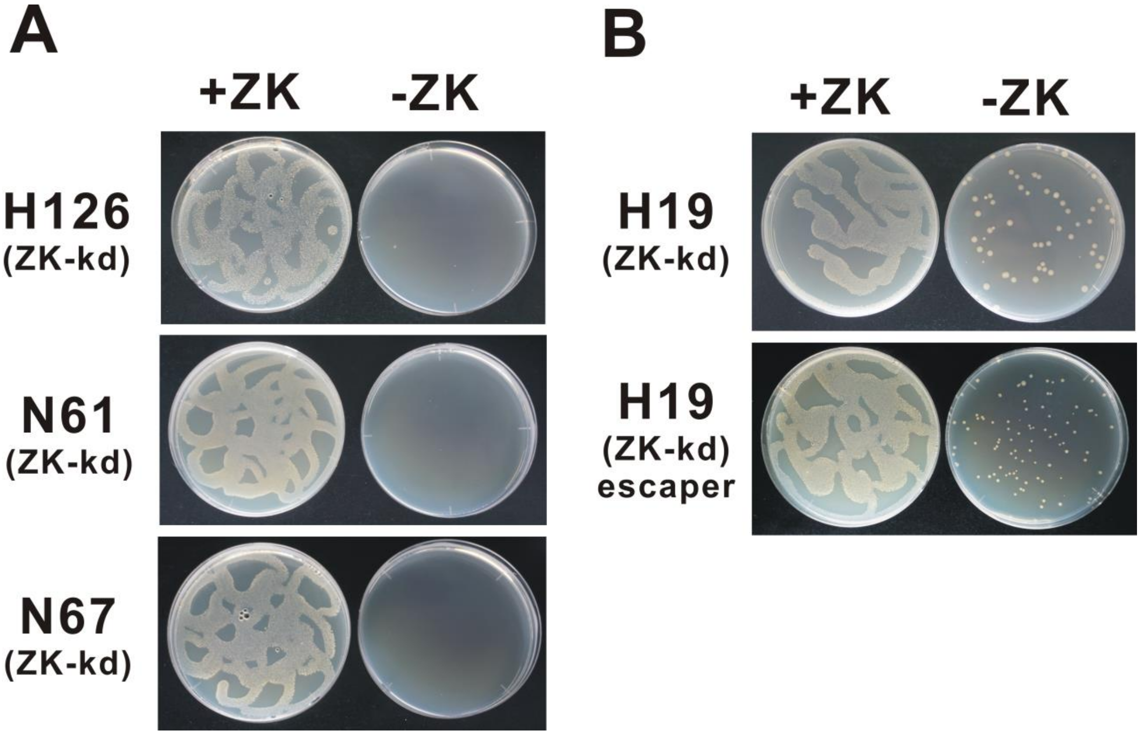

Supplementary Figure 5 Transformation of the ZK-regulated *kid-kis* and *ccdB-ccdA* toxin-antitoxin systems into *E. coli* clinical isolates.

Plasmid A-3 was transfected into *E. coli* clinical isolates. Transformants were successfully generated for 4 strains, H19, H126, N61 and N67. Colony formation was evaluated in the presence and absence of 3 mM ZK. Uneven colony distribution is an artificial phenomenon caused by uneven inoculation. See also Materials and Methods. (A) H126, N61 and N67. (B) H-19. Note a higher emergence of escapers even in the absence of ZK. Top panel, the first generation after the plasmid A-3 transformation. Bottom panel, a recultured escaper isolated from the first generation.

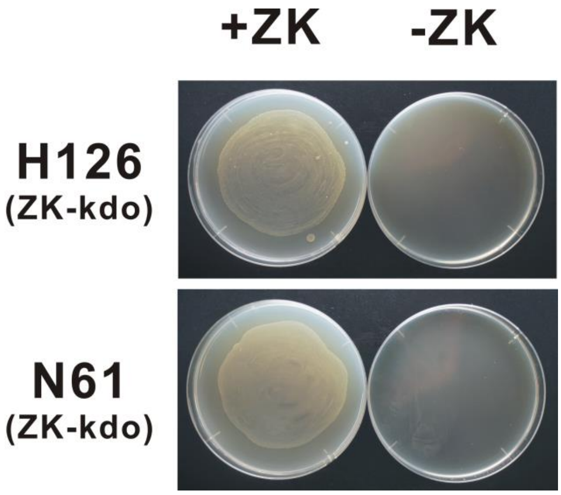

Supplementary Figure 6 Additional transformation of the ZK-regulated *colE3-immE3* toxin-antitoxin system.

Plasmid C-1 was additionally transformed into the resulting strains shown in Supplementary Figure 5. Growth was evaluated in the presence and absence of 3 mM ZK.

Supplementary Table 1. Growth rates.

Growth curves were determined for some ZK-auxotrophic strains and their vector control strains. Doubling times were calculated during the logarithmic growth phase. Growth rates were shown as a relative value (vector control = 1).

	Doubling time (min)		Relative growth rate (V/T)
	Tested strain (T)	Vector control strain (V)	
E. coli (laboratory strain)			
BL21-AI(ZK-o)	82.0	73.0	0.89
BL21-AI(ZK-kd)	72.3	48.9	0.68
BL21-AI(ZK-kdo)	100.7	73.0	0.72
E. coli (clinical isolate)			
N61（ZK-kdo）	60.1	38.8	0.65
S. enterica			
χ3306 (ZK-k)	43.6	26.9	0.62
χ3306 (ZK-kd)	29.1	26.9	0.93

Supplementary Table 2. Disease score

Disease Score	Characterization	Clinical signs
0	No clinical signs	-
1	Mild clinical signs[a]	Ruffled fur
2	Moderate clinical signs	Ruffled fur plus, lethargy, hunched posture, decreased activity
3	Severe clinical signs	Paresis, paralysis, tremor, shivers, ataxia, rigidity, coma
4	Death	-

[a] Mice that exhibited only mild clinical signs, showed no further signs of disease, and recovered until the end of the experiment were not considered ill.
